# Antimicrobial resistance profiles and molecular epidemiology of *Klebsiella pneumoniae* isolates from Scottish bovine mastitis cases

**DOI:** 10.1017/S0950268824001754

**Published:** 2025-01-17

**Authors:** Jolinda Pollock, Geoffrey Foster, Katrina Henderson, Jennifer Bell, Michael R. Hutchings, Gavin K. Paterson

**Affiliations:** 1 Microbiology Department, SRUC Veterinary Services, Edinburgh/Inverness, UK; 2 Animal and Veterinary Sciences, SRUC, Edinburgh, UK; 3 Royal (Dick) School of Veterinary Studies, University of Edinburgh, Edinburgh, UK; 4 Roslin Institute, University of Edinburgh, Edinburgh, UK

**Keywords:** Klebsiella, mastitis, AMR, MALDI-TOF, WGS

## Abstract

*Klebsiella pneumoniae* are opportunistic pathogens which can cause mastitis in dairy cattle. *K. pneumoniae* mastitis often has a poor cure rate and can lead to the development of chronic infection, which has an impact on both health and production. However, there are few studies which aim to fully characterize *K. pneumoniae* by whole-genome sequencing from bovine mastitis cases. Here, *K. pneumoniae* isolates associated with mastitis in dairy cattle were identified using matrix-assisted laser desorption/ionisation time-of-flight mass spectrometry (MALDI-TOF MS) and whole-genome sequencing. Furthermore, whole-genome sequence data were used for phylogenetic analyses and both virulence and antimicrobial resistance (AMR) prediction, in parallel with phenotypic AMR testing. Forty-two isolates identified as *K. pneumoniae* were subject to whole-genome sequencing, with 31 multi-locus sequence types being observed, suggesting the source of these isolates was likely environmental. Isolates were examined for key virulence determinants encoding acquired siderophores, colibactin, and hypermucoidy. The majority of these were absent, except for *ybST* (encoding yersiniabactin) which was present in six isolates. Across the dataset, there were notable levels of phenotypic AMR against streptomycin (26.2%) and tetracycline (19%), and intermediate susceptibility to cephalexin (26.2%) and neomycin (21.4%). Of importance was the detection of two ESBL-producing isolates, which demonstrated multi-drug resistance to amoxicillin-clavulanic acid, streptomycin, tetracycline, cefotaxime, cephalexin, and cefquinome.

## Introduction

Mastitis is a prevalent and important disease in cattle worldwide, which has significant health, welfare, and economic impacts [1–3]. Mastitis control programmes have primarily focussed on contagious mastitis pathogens and not environmental opportunists [4]. Environmental mastitis can be caused by a large range of bacteria, with one of the most predominant and clinically significant being *K. pneumoniae* [4–6]. *K. pneumoniae* is one of the most common coliforms causing clinical bovine mastitis, but has been found to be one of the most damaging when considering milk production [7], treatment costs [4], and mortality rate [5]. *K. pneumoniae* mastitis has a poor cure rate after antimicrobial treatment [5, 8, 9], and although it is commonly an environmental opportunist, lateral spread from diseased to healthy cattle is possible [10].

Antimicrobial resistance (AMR) is an ongoing concern in both animal and human health settings. Due to the ubiquitous nature of *K. pneumoniae*, there are varied reports on the AMR potential in these strains. In some previous work, it has been shown that *K. pneumoniae* isolates from milk showed low levels of AMR [11–13]. However, there have been reports of multi-drug resistant *K. pneumoniae* isolated from bovine mastitis cases from several countries [14–16], including the United Kingdom [17], most of which are extended-spectrum beta-lactamase producers. More concerning reports have also emerged describing carbapenem resistance in *K. pneumoniae* isolates from cattle milk [18, 19], highlighting the importance of ongoing disease and AMR surveillance.

Whole-genome sequencing, in combination with diagnostic laboratory identification and antimicrobial sensitivity testing (AST), gives a comprehensive insight into bacterial epidemiology, virulence potential, and AMR. Here, these methodologies are used to characterise *K. pneumoniae* isolates from mastitis cases in Scotland between 2009 and 2021.

## Materials and methods

### Isolates and primary identification

Forty-two isolates, previously identified as *K. pneumoniae* from clinical or sub-clinical mastitis cases that had been stored on glycerol beads (SRUC Veterinary Services’ Pathogen Bank) at −80 °C, were revived by culture on Columbia agar supplemented with sheep blood (Oxoid, United Kingdom), incubated aerobically at 37°C for 24 h. Isolates were obtained from cattle showing signs of sub-clinical or clinical mastitis between 2009 and 2021. Further details of these isolates and a summary of clinical histories are available in Supplementary Table S1.

Isolates were identified on initial isolation using API 20E strips (Biomerieux, France) according to the manufacturer’s instructions. As part of this study, isolates were also identified post hoc by matrix-assisted laser desorption/ionisation time-of-flight mass spectrometry using a Microflex LT/SH Biotyper (MALDI-TOF MS) (Bruker, Germany). Briefly, single and well-isolated colonies were spotted directly in duplicate onto Disposable MBT Biotarget 96 target plates (Bruker, Germany) using the direct transfer method. Spots were then overlaid with 1 μl of α-Cyano-4-hydroxycinnamic acid (HCCA) matrix and allowed to dry, prior to transfer to the instrument. The generated spectra were then subject to best match identification and confidence scoring, with scores of ≥2.00 deemed as good identifications.

### Whole-genome sequencing

Whole-genome sequencing was performed by Microbes NG (University of Birmingham, UK), as described previously [20]. Briefly, genomic DNA was extracted using solid-phase reversible immobilisation beads, and genomic DNA libraries were prepared using the Nextera XT Library Prep Kit (Illumina) following the manufacturer’s protocol with the following modifications: input DNA is increased by twofold, and PCR elongation time is increased to 45 s. Libraries were sequenced using Illumina sequencers (HiSeq/NovaSeq) using a 250-bp paired-end protocol. Reads were trimmed using Trimmomatic version 0.30 [21], using a sliding window quality cut-off of 15. Genome assembly was carried out de novo using SPAdes, version 3.7, with default parameters for 250 bp Illumina reads [22] and annotated by the National Center for Biotechnology Information (NCBI) Prokaryotic Genome Annotation Pipeline v6.6 [23]. Contigs <200 bp were manually removed, and contaminant sequences were automatically removed during upload to NCBI via the NCBI Foreign Contamination Screen [24]. Genome assemblies were uploaded and analysed using Pathogenwatch [25] v22.1.1 (https://pathogen.watch/) and Kleborate v2.3.0 [26] incorporated therein. Resultant trees were annotated using the Interactive Tree of Life (iTOL) [27, 28].

### Antimicrobial sensitivity testing

AST using nine antimicrobials – amoxicillin–clavulanic acid 30 μg, ertapenem 10 μg, streptomycin 10 μg, enrofloxacin 5 μg, tetracyline 30 μg, cefotaxime 5 μg, cephalexin 30 μg, neomycin 30 μg, and cefquinome 30 μg (Oxoid, United Kingdom). The EUCAST disc diffusion method was used on Mueller–Hinton Agar (Oxoid, United Kingdom) using EUCAST [29], CLSI Vet [30], and CASFM Vétérinaire [31] guidelines. Namely, antimicrobial sensitivity was assessed for amoxicillin–clavulanic acid, ertapenem, streptomycin, enrofloxacin, tetracycline, cefotaxime, cephalexin, neomycin, and cefquinome. Isolates that were highlighted as potential extended-spectrum beta-lactamase (ESBL) producers by AST were subject to ESBL screening using a commercially available ESBL, AmpC, and carbapenemase activity kit (MAST, United Kingdom).

## Results

### Species identification of bovine mastitis isolates

Forty-two bovine mastitis isolates initially identified by MALDI-TOF MS and API 20E as being *K. pneumoniae* were subject to whole-genome sequencing by Illumina technology, and the resultant assemblies were analysed via Kleborate. The 42 isolates were confirmed, based on Kleborate and Type Strain Genome Server analysis [32] of their genome sequence, as being *K. pneumoniae* (Supplementary Table S1).

### Antimicrobial sensitivity testing

All isolates were susceptible to ertapenem and enrofloxacin (Table 1). Of note were two ESBL-producing isolates, which showed multi-drug resistance to amoxicillin–clavulanic acid, streptomycin, tetracycline, cefotaxime, cephalexin, and cefquinome. Excluding the ESBL-producing isolates, there was a prevalence of streptomycin (26.2%) and tetracycline (19.0%) resistance, with a notable number of isolates showing intermediate susceptibility to cephalexin (26.2%) and neomycin (21.4%).

**Table 1.** Number and percentage of studied isolates which showed sensitive, intermediate, or resistant susceptibility profiles against the listed antimicrobial agents

	Susceptibility profile		
Antibiotic	Sensitive	Intermediate	Resistant
Cefquinome	40 (95.2%)	0 (0%)	2 (4.8%)
Cefotaxime	40 (95.2%)	0 (0%)	2 (4.8%)
Streptomycin	28 (66.7%)	3 (7.1%)	11 (26.2%)
Tetracycline	34 (81.0%)	0 (0%)	8 (19.0%)
Amoxicillin-clavulanic acid	39 (92.9%)	N/A	3 (7.1%)
Cephalexin	29 (69.0%)	11 (26.2%)	2 (4.8%)
Neomycin	33 (78.6%)	9 (21.4%)	0 (0%)
Ertapenem	0 (0%)	N/A	0 (0%)
Enrofloxacin	0 (0%)	0 (0%)	0 (0%)

### Molecular characterisation of isolates

**Figure 1.**
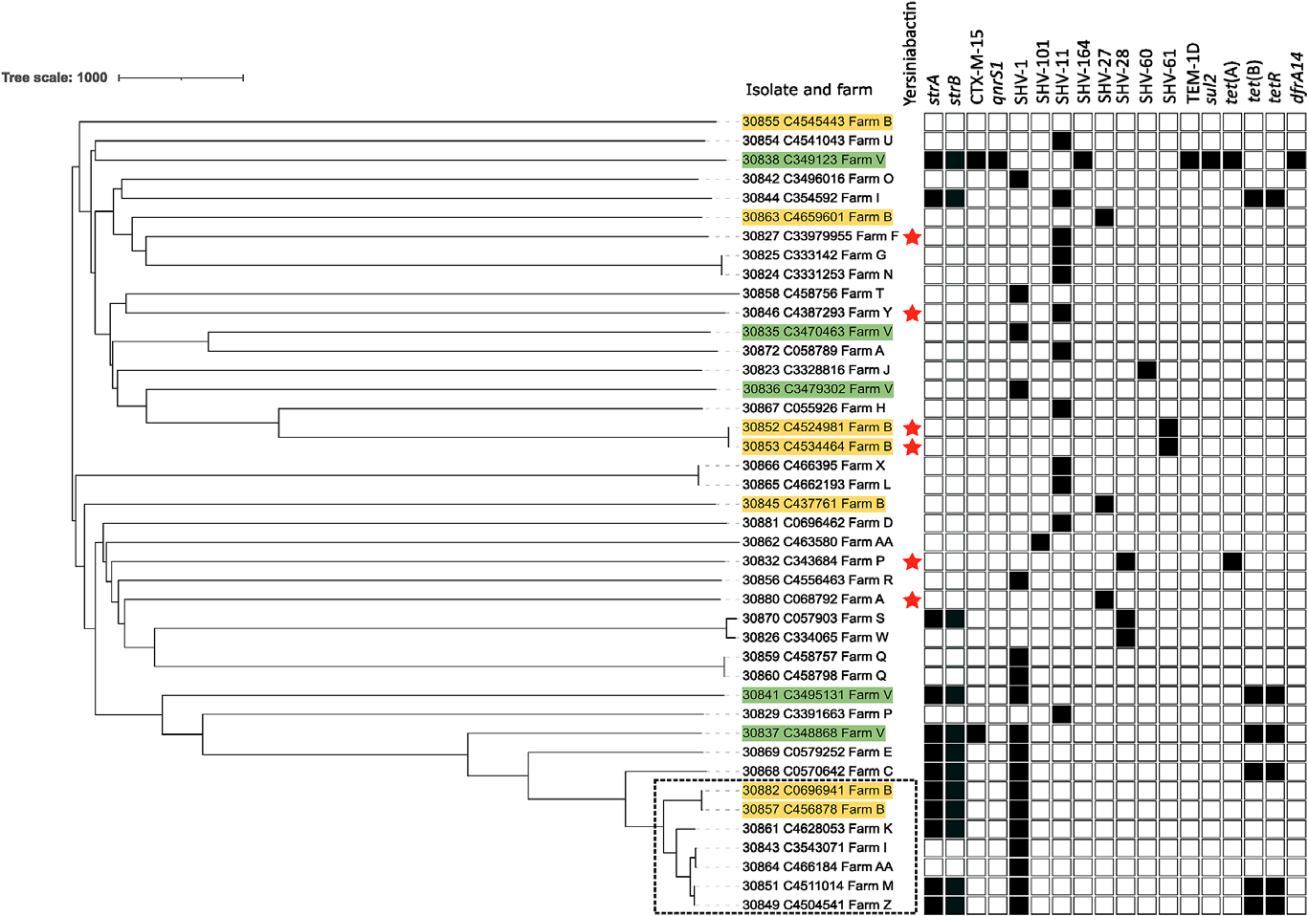
Mid-point rooted, neighbour-joining tree showing phylogenetic relationships among *K. pneumoniae* mastitis isolates from Scotland. Tree constructed using pairwise SNP distances across 1972 *K. pneumoniae* core genes and produced by Pathogenwatch [25]. Multiple isolates came from Farms B (7 isolates) and V (5 isolates), and these are highlighted in green and yellow, respectively. ST107 isolates are highlighted in a black box. The presence of yersiniabactin is denoted by red star and antimicrobial resistance genes are shown by squares as being either present (filled black square) or absent (empty square).

The 42 *K. pneumoniae* isolates originated from 28 dairy herd premises, 7 isolates came from the same farm, 4 from another farm, 2 from five other farms with the rest being single isolates from the remaining 21 premises. Their genome size varied from 5236065 to 5764556 Mb with a mean of 5454345 Mb (Supplementary Table S2). The G + C content ranged from 56.6 to 57.6 mol% with a mean of 57.2 mol% (Supplementary Table S2). The 42 isolates belonged to 31 different multi-locus sequence types (https://bigsdb.pasteur.fr/ ) each of which was represented by one or two isolates with the exception of seven ST107 isolates. One isolate (30881_C069646/2) belonged to a new sequence type – ST6855. The mean pairwise single nucleotide polymorphism (SNP) distance between isolates was 9234 SNPs (range 0–10665) (Supplementary Table S2). There were seven pairs of identical/near-identical isolates (separated by 0–7 SNPs). In four of these cases, the two isolates came from different farms (Figure 1).

### AMR determinants

Ten different acquired AMR genes were present among the 42 isolates with the aminoglycoside phosphotransferases *strA*/*APH(3″)-Ib* and *strB*/*APH(6)-Id* being the most abundant (present in 12 isolates) (Figure 1). All but one isolate carried the chromosomally encoded SHV β-lactamase with eight different alleles being present. None of these alleles carry known class-modifying mutations, i.e. conferring resistance to extended-spectrum β-lactams or β-lactamase inhibitors [26]. Two isolates, 30837_C348868 and 30838_C349123, were ESBL producers and encoded CTX-M-15. Despite originating from the same premises (Supplementary Table S2), these isolates are phylogenetically unrelated based on core genome SNPs (Figure 1).

### Virulence determinants

Isolates were examined for the presence of the virulence-related genes encoding acquired siderophores (yersiniabactin, salmochelin, aerobactin), the genotoxin colibactin, the hypermucoidy locus *rmpADC*, and alternative hypermucoidy marker gene *rmpA2.* These were absent from all isolates except for *ybST* (encoding yersiniabactin) which was present in six isolates, of which only two are closely related (Figure 1 and Supplementary Table S1).

### Prevalence of ST107

**Figure 2.**
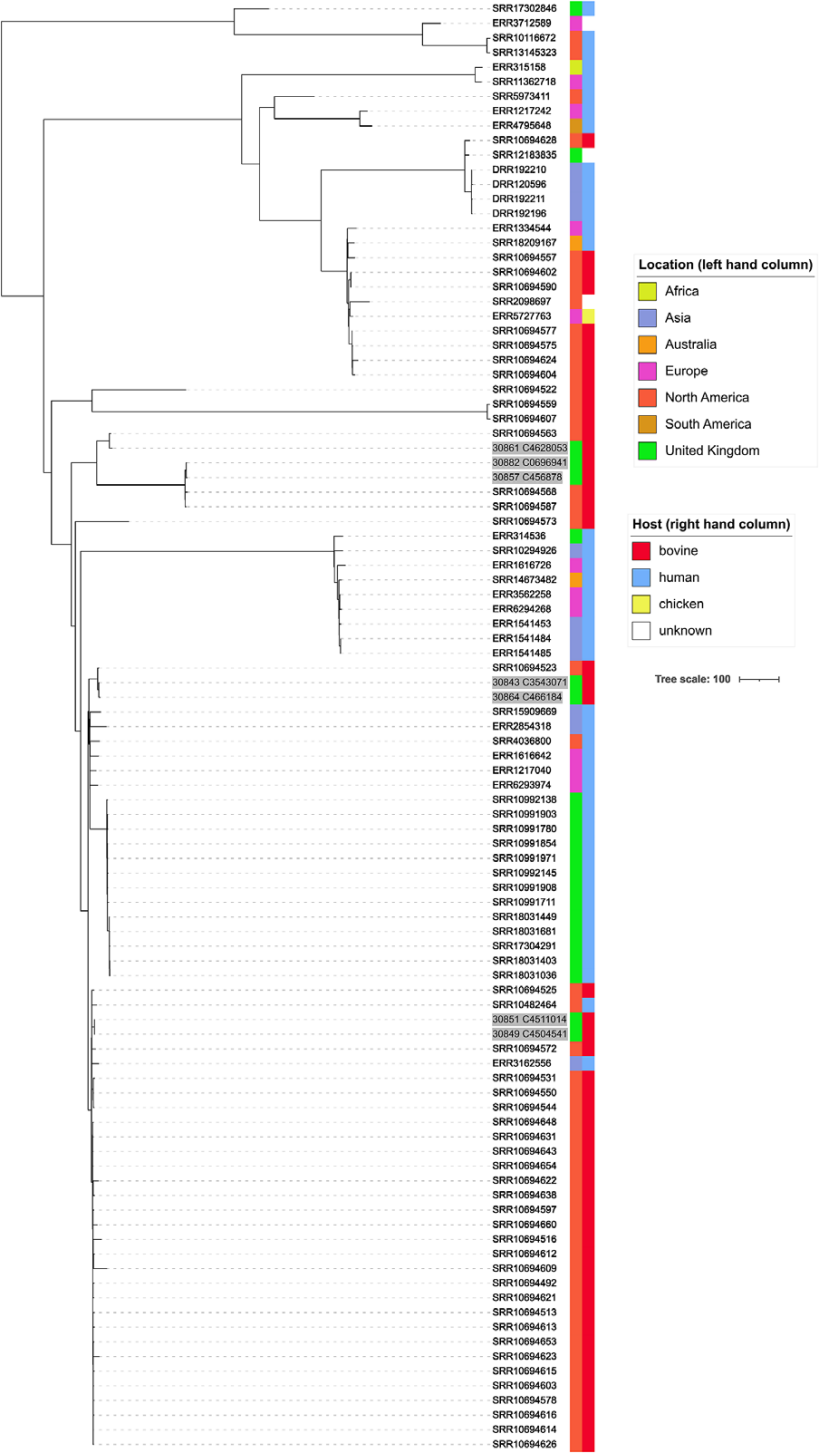
Mid-point rooted, neighbour-joining tree showing phylogenetic relationships among international isolates *K. pneumoniae* ST107. Tree constructed using pairwise SNP distances across 1972 *K. pneumoniae* core genes and produced by Pathogenwatch [25]. Geographical location of origin and host are indicated by a coloured key. Labels of study isolates are shaded grey, and UK is differentiated from Europe for the purposes of this figure.

The most common multi-locus sequence type was ST107, with seven isolates being detected from six farms. These were investigated further by phylogenetic comparison with all 92 ST107 isolate genomes available from Pathogenwatch (accessed 17/01/2023) (Supplementary Table S3). Metadata showed that these 92 isolates came from 17 countries and from the continents of Australia (2 isolates), Africa [1], Asia [11], Europe [27], North America [50], and South America [1]. These isolates were from cattle (44 isolates), humans (44 isolates), chickens [1], and unknown sources [3]. The resultant tree (Figure 2) showed that isolates from this study did not all cluster together within the ST107 global population. While each isolate was always most closely related to another study isolate, they were all also closely related (10–19 SNPs) to three bovine isolates from North America (Figure 2).

## Discussion

*Klebsiella pneumoniae* is a well-studied pathogen in the context of human infection [33, 34], but less research has been carried out on bovine mastitis isolates [35]. The aim of this study was to carry out a systematic analysis of *K. pneumoniae* isolates causing sub-clinical and clinical mastitis in Scotland, focussing on comprehensive phenotypic identification, antimicrobial sensitivity, molecular characteristics, and population structure.

First, there was marked diversity in *K. pneumoniae* isolates by MLST, identifying 31 sequence types among the 42 strains. This finding has been mirrored in previous work, which also showed high diversity in *K. pneumoniae* genomes [35, 36]. *Klebsiella* species are common inhabitants on the teat skin of dairy cattle [37]; thus, the sample collection method may have a part to play in the diversity of *Klebsiella* species found in milk samples [38] and could also be suggestive of an environmental source of infection [13]. Despite the heterogeneity, the same strains have been implicated in infections on the same units in this study, and in previous mastitis studies [39, 40], which may suggest a common source of infection.

Second, despite the diversity in this dataset by MLST, 7 of 42 isolates belonged to ST107. The frequency of the remaining STs was two or less, highlighting the diversity of *K. pneumoniae* strains on-farm. These ST107 strains were observed across six farms. These isolates were included in a meta-analysis with other ST107 strains globally, which appeared to be more related overall to other bovine isolates than other species. Indeed, other studies have found that ST107 is predominant [35, 36, 41, 42]. Other work has shown that ST107 are one of the most common strains associated with clinical mastitis [43], which could potentially suggest lateral spread of the infection rather than being strictly environmental [10]. This could be of clinical significance since it becomes more of a risk that infection could occur from other sources, such as milking equipment, increasing the likelihood of a herd-level infection rather than sporadic infection.

Third, virulence factors were considered using whole-genome sequencing data. The study isolates were examined for genes encoding yersiniabactin, salmochelin, aerobactin, colibactin, the hypermucoidy locus *rmpADC* and the alternative hypermucoidy marker gene *rmpA2.* Most of these virulence factors were not observed, except for *ybST*, which encodes yersiniabactin. This gene was found in six of the study isolates, from five different STs (ST37, ST458, ST111, ST1109 (*n* = 2), and ST976). Previous work has highlighted that many of the virulence genes mentioned previously are less common in bovine isolates in comparison to human isolates [43–45] and are associated with increased virulence. However, we do not have the data resolution as part of this study to determine the role of yersiniabactin in virulence.

Fourth, the AMR phenotypes were well predicted by known AMR genes. Ten different acquired AMR genes were observed across the dataset, with the aminoglycoside phosphotransferases *strA*/*APH(3″)-Ib* and *strB*/*APH(6)-Id* being the most prevalent (present in 28.6% of isolates). Indeed, 26.2% of the study isolates showed phenotypic resistance to streptomycin and 21.4% showed intermediate sensitivity to neomycin (both aminoglycoside antibiotics). Phenotypic resistance to tetracycline was also prevalent (19% of isolates), with *tetA* being observed in 4.8% of isolates, and both *tetB* and *tetR* genes being observed in pairs across 14.3% of isolates. Intermediate sensitivity to cephalexin was observed in over a quarter of isolates (26.2%), and all bar one isolates carried the chromosomally encoded SHV β-lactamase, showing eight different alleles. None of the eight alleles carry known class-modifying mutations linked to resistance to extended-spectrum β-lactams or β-lactamase inhibitors.

Finally, linked to the above, we identified two suspected ESBL-producing isolates from the same premises (encoding CTX-M-15) in this dataset by whole-genome sequencing, which was confirmed phenotypically. These isolates showed multi-drug resistance to amoxicillin–clavulanic acid, streptomycin, tetracycline, cefotaxime, cephalexin, and cefquinome. Critically, no study isolates showed carbapenemase activity, but there has been a series of recent work reporting carbapenem resistance in bovine mastitis isolates [18, 19, 46, 47], highlighting the importance of disease and AMR surveillance work.

## Conclusions

To conclude, the genetic diversity in the described *K. pneumoniae* isolates is high with the most dominant ST107 potentially linked to lateral spread. The number of well-described virulence genes present was low, with *ybST* (encoding yersiniabactin) being observed in a small number of isolates. Higher levels of AMR to aminoglycosides and tetracycline were present, with two ESBL-producing isolates being observed. This study highlights the importance of monitoring bacterial isolates in clinical cases considering concerns about multi-drug resistance. This work should inform more extensive future studies including a larger number of farm units over a period of time.

## Supporting information

Pollock et al. supplementary material 1 Pollock et al. supplementary material

Pollock et al. supplementary material 2 Pollock et al. supplementary material

Pollock et al. supplementary material 3 Pollock et al. supplementary material

## Data Availability

Whole-genome sequence data are available on Genbank (BioProject PRJNA943144, accession numbers and genome descriptive statistics are provided in Supplementary Table S3).

## Supplementary material

The supplementary material for this article can be found at http://doi.org/10.1017/S0950268824001754.
